# Methicillin-resistant *Staphylococcus pseudintermedius*: epidemiological changes, antibiotic resistance, and alternative therapeutic strategies

**DOI:** 10.1007/s11259-024-10508-8

**Published:** 2024-08-21

**Authors:** Francesca Paola Nocera, Luisa De Martino

**Affiliations:** Department of Veterinary Medicine and Animal Production, University of Naples “Federico II”, Naples, Italy

**Keywords:** Methicillin-resistant *S. pseudintermedius*, Epidemiology, Multidrug resistance, Alternative therapies

## Abstract

*Staphylococcus pseudintermedius* is a major opportunistic bacterial pathogen that belongs to the skin and mucosal microbiota of the dog. Since its global emergence around 2006, multidrug - methicillin-resistant *S. pseudintermedius* (MRSP) clones have become endemic worldwide. MRSP strains pose a significant threat to animal health and make antimicrobial therapy difficult due to their typical multidrug resistance phenotypes. The difficulty to treat MRSP infections using the current antimicrobials licensed for veterinary use has intensified research efforts to develop new treatment strategies and alternative anti-infective approaches to conventional antimicrobial therapy. The present narrative review outlines the latest changes in the epidemiology of MRSP with focus on the geographical distribution variability and antimicrobial resistance profiles in the main MRSP lineages. It also provides an overview of the effectiveness of currently available antimicrobials and the status of anti-infective alternatives to conventional antimicrobials.

Recent studies have reported notable changes in the population structure of MRSP, with the emergence of new epidemic lineages, such as ST258, ST123, ST496, and ST551 in European countries and ST45, ST181, ST258, ST496 in non-European countries, which partly or totally replaced those that were initially prevalent, such as ST71 in Europe and ST68 in the US. Due to methicillin resistance often associated with the resistance to a broader number of antimicrobials, treating canine MRSP skin infection is challenging. Several alternative or supplementary treatment options to conventional antibiotics, especially for topical treatment, such as a novel water-soluble hydroxypyridinone-containing iron-chelating 9 kDa polymer (DIBI), antimicrobial peptides (AMPs), nanoparticles, and bacteriophages seem to be particularly interesting from a clinical perspective.

## Introduction

*Staphylococcus pseudintermedius* is the major coagulase-positive staphylococcal species that colonizes and infects dogs. It can be isolated from the nares, oral mucosa, pharynx, forehead, groin, and anus of healthy dogs (Bannoehr and Guardabassi 2012). Under particular conditions, such as allergy, endocrine or immune disorders, parasite infestation, this opportunistic pathogen can take advantage of the weakened host defenses and cause infection, including pyoderma, otitis, abscesses, surgical site and urinary tract infection (Bannoehr and Guardabassi 2012). Although *S. pseudintermedius* can also be found in cats and horses, it is not as prevalent as in dogs, where it colonizes 77–90% of healthy animals (Perreten et al. 2010; van Duijkeren et al. 2011). In addition, *S. pseudintermedius* has been associated with several cases of human colonization and infections, primarily resulting from close interactions between humans and their pet dogs (Blondeau et al. 2020; Small et al. 2021; Asleh et al. 2022).

*S. pseudintermedius* isolates were generally susceptible to penicillase-insensitive β-lactams, which include antibiotics widely used in small animal veterinary practice such as amoxicillin/clavulanic acid, cephalexin, and cefazolin. Therapy has become problematic because of the global emergence of methicillin-resistant *S. pseudintermedius* (MRSP), which has been increasingly reported in dogs since 2006 (Krapf et al. 2019). MRSP strains are considered by definition as resistant to any β-lactams and often exhibit resistance to multiple classes of antimicrobials other than β-lactams, therefore limiting the possible treatment options. Multidrug resistance in MRSP may virtually comprise all antimicrobial classes approved for veterinary use including tetracyclines, macrolides, lincosamides, aminoglycosides, streptogramins, trimethoprim-sulfamethoxazole, and fluoroquinolones (Perreten et al. 2010). Biofilm-associated infections caused by MRSP pose additional challenges as biofilms provide protection against antimicrobial agents and the immune system, leading to persistent infections (Osland et al. 2012). This situation has induced veterinarians to use antimicrobials that are only approved for human use such as amikacin, rifampicin, linezolid, which are considered by the World Health Organization as ‘critically important antimicrobials” (WHO, 2018) and should be preserved for treatment of multidrug-resistant bacterial infections in human medicine. This denotes a true therapeutic dilemma, due both to potential drug toxicities (amikacin and rifampicin) and ethical considerations (vancomycin and linezolid) (Morris et al. 2017). According to the Antimicrobial Advice Ad Hoc Expert Group (AMEG) categorization of antibiotics for use on animals, these antibiotics belong to category A (“Avoid”) and their use is not authorized in food producing animals, but their administration is allowed in companion animals only under exceptional circumstances (EMA 2020). Recently, the European Union has introduced a new regulation defining a list of antimicrobials solely for human use (Commission Implementing Regulation EU 2022/1255 2022), making them unavailable as reserve drugs for life-threatening MRSP infections in animals. Among them, such a list includes vancomycin, linezolid, and fosfomycin.

The effectiveness of the currently available antimicrobials for MRSP infections varies depending on the clonal lineage and the specific strain’s resistance profile. A systematic review was published in 2016 and indicated significant differences in geographical distribution and antimicrobial resistance of the main MRSP clonal lineages (Pires Dos Santos et al. 2016). The present review outlines the recent epidemiological changes with focus on the consequences on antimicrobial chemotherapy, evaluating the effectiveness of currently available antimicrobials against the major MRSP lineages. It also examines the potential of novel alternatives to conventional antimicrobials such as iron-chelating agents, antimicrobial peptides, nanoparticles, bacteriophages.

## Materials and methods

This narrative review is intended to describe and synthesize the available literature on the following topics: *i*) MRSP population structure variations after 2016, *ii*) antimicrobial resistance changes in the most common MRSP Sequence Types (STs) spread globally, *iii*) overview of the most recent studies on new antimicrobial treatment strategies and alternatives to antibiotics.

The data of this narrative review was obtained from articles published in English performing a comprehensive search through PubMed database, using the following keywords: methicillin-resistant *S. pseudintermedius*; Sequence Types; antimicrobial resistance patterns; new alternative therapeutic approaches. The search was limited to articles between the timeframe 2010–2023. A total of 350 articles were initially identified and only 86 of them were included in this study, containing at least two of the above-mentioned keywords in the title and/or abstract. Furthermore, the open-access MLST database for *S. pseudintermedius* (https://pubmlst.org/spseudintermedius/) was also consulted to analyze the evolutionary changes of the MRSP population structure.

### MRSP molecular epidemiology and population structure

**Fig. 1:**
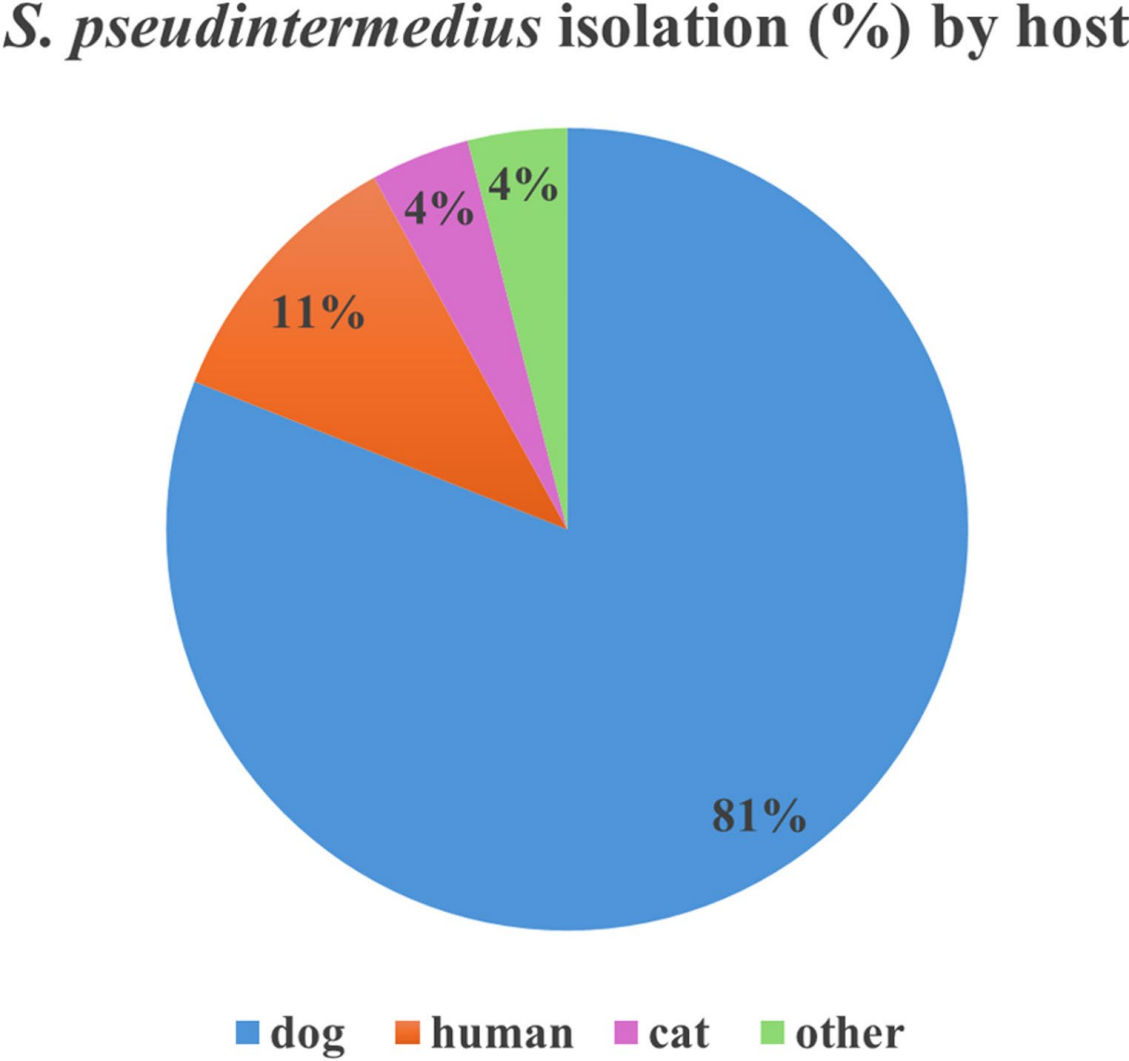
*S. pseudintermedius* isolation in different hosts by using data from https://pubmlst.org/spseudintermedius/

Over the last ten years, the population structure and the high genetic diversity of *S. pseudintermedius* have been better defined by the introduction of the species-specific multilocus sequence typing MLST-7, developed by Solyman et al. (2013). The publicly available MLST database for *S. pseudintermedius* offers the possibility to characterize, besides the specific structure of this bacterial population, its long-term epidemiological trends worldwide (Jolley et al. 2018). To date, the database comprises records of 517 sequence types (STs) and 3,110 isolates. Most of the submissions are from Europe (36.43%), followed by North America (34.41%), Asia (19.71%), South America (5.85%), Oceania (2.41%) and Africa (1.13%). The database includes 38.3% and 61.7% submissions of MRSP and methicillin-susceptible *S. pseudintermedius* (MSSP) isolates, respectively. As reported in Fig. 1, humans are the second most affected host species after dogs, based on PubMLST *S. pseudintermedius* database data (Jolley et al. 2018).

MLST allelic data analysis has revealed that the population structure of *S. pseudintermedius* is highly diverse because of frequent recombination events (Pires Dos Santos et al. 2016). MRSP strains evolve in phylogenetically distinct clonal lineages through multiple autonomous SCC*mec* and Tn5405-like elements acquisitions, or gyrase mutations, determining a selection of resistant clones and their clonal expansion (McCarthy et al. 2015). Some geographical patterns are evident in the distribution of MRSP clonal lineages around the world. Among the lineages that first acquired methicillin resistance, ST71, originally described as the epidemic European clone (Haenni et al. 2014; Damborg et al. 2016; Ventrella et al. 2017; Menandro et al. 2019; Nocera et al. 2020c), is now widespread worldwide, including North and South America (Quitoco et al. 2013; Penna et al. 2022; Viegas et al. 2022), New Zealand (Nisa et al. 2019) and Japan (Ishihara et al. 2016), while ST68, previously described as the epidemic North American clone, has been frequently reported also in Europe (Perreten et al. 2010; Ruiz-Ripa et al. 2021). Differently from ST71, ST68 has not spread yet in Asian countries (Ishihara et al. 2016), where ST45 and ST112 were reported as the prevalent lineages by the systematic review by Pires Dos Santos et al. (2016), with the current addition of ST181 as displayed in *S. pseudintermedius* MLST database (https://pubmlst.org/spseudintermedius/).

The epidemiological landscape of MRSP has further evolved in recent years with the global spread of other lineages such as ST45, ST112, ST169, ST181, ST258, ST261, ST265, ST496, and ST551 (Kjellman et al. 2015; Duim et al. 2016; Ishihara et al. 2016; Ventrella et al. 2017; Grönthal et al. 2017; Kizerwetter-Świda et al. 2017; Bergot et al. 2018; Nocera et al. 2020c; Silva et al. 2021; Ferrer et al. 2021). Various studies have reported an increasing prevalence of ST258, which seems to be replacing ST71 in Northern European countries (Kjellman et al. 2015; Damborg et al. 2016; Duim et al. 2016; Grönthal et al. 2017) and in France (Bergot et al. 2018). However, a high genotypic diversity among MRSP isolated from healthy and diseased dogs was detected in Denmark (Damborg et al. 2016) and in Norway (Kjellman et al. 2015). ST258 has been also described in Southern Italy, even though here ST71 still represents the dominant clone (Ventrella et al. 2017; Nocera et al. 2020c). Differently from ST71, ST258 appears to be more susceptible to veterinary-licensed antibiotics (Damborg et al. 2016; Duim et al. 2016; Grönthal et al. 2017). Bergot et al. (2018) analyzed the phylogeny of the French ST71, ST258, ST496, highlighting that ST71 and ST496 populations are highly homogenous, whilst ST258 population is extremely heterogeneous. This was also confirmed by subsequent studies (Kjellman et al. 2015; Pirolo et al. 2023). The reasons for the successful dissemination of ST258 and the associated clonal complex (CC258) in Northern Europe remain unclear. However, the possibility to exchange antimicrobial resistance genes, carried by mobile genetic elements (McCarthy et al. 2015), as well as genes associated with other virulence factors, such as biofilm production (Osland et al. 2012) or the ability to evade host’s immune response thanks to the production of the protein A able to bind the Fc region of immunoglobulins (Balachandran et al. 2018) may favor a successful selection and, consequently, the spread of some major clonal lineages rather than others, becoming a relevant challenge for both veterinary and human medicine.

In Portugal, until 2016, ST71 was still the dominant clone, then Silva et al. (2021) highlighted that ST123 was the most frequent clone among the MRSP isolates from canine pyoderma. Hence, a shift in the Portuguese country might have been occurred, probably due to the higher evolutionary rate of ST123 compared to ST71 (Silva et al. 2021). While ST71 has been detected mainly in MRSP strains, and only in one case in a MSSP strain isolated from a healthy dog in Japan as documented on Pub-MLST *S. pseudintermedius* (https://pubmlst.org/spseudintermedius/), ST123 has been recovered among both MRSP and MSSP strains (https://pubmlst.org/spseudintermedius/).

In Poland, a notable shift indicating changes in MRSP population structure has been reported since 2016. In fact, ST71 started to be replaced by ST551, identified in most strains isolated from canine population (Kizerwetter-Świda et al. 2017).

Outside Europe, the newly emerging ST496 has been identified for the first time in Australia and described as one of the main MRSP clones circulating in Sidney (Worthing et al. 2018). Shortly thereafter, ST496 has been found also in Europe, precisely in France (Bergot et al. 2018) and Italy (Nocera et al. 2020c). In a study carried out in US, Phophi et al. (2023) highlighted the changes occurred to the MRSP population structure in the last decade. Precisely, the US predominant STs, namely ST68, ST71, and ST84, have been substituted by ST45, ST155, ST181, ST496, and ST551, even though no common shared ancestor was found (Phophi et al. 2023). Furthermore, in the study carried out by Sawhney et al. (2023) in the American Midwest, the most frequently identified STs among canine and human *S. pseudintermedius* isolates were the previously described ST759, ST181, and ST923, together with the first identification of ST862 in the American Midwest.

On the contrary, for MRSP from Asia, ST45 and ST181 remain the dominant STs, as reported on publicly genome depository (https://pubmlst.org/spseudintermedius/). However, in recent years a great variety of STs, including many completely new ones but without a predominant type, has been recorded both in Asia (Han et al. 2018; Lee et al., 2020) and Africa (Youn et al. 2014).

It has been hypothesized that the successful spread of certain MRSP clones may be linked to advantageous fitness properties such as the ability to form biofilm (Osland et al. 2012). Indeed, isolates classified as ST71 demonstrated a significantly higher capacity for biofilm production compared to other isolates (Osland et al. 2012; Andrade et al. 2022). Abundant biofilm formation probably provides a competitive advantage, contributing to its notable success as a pathogen in certain clones. This is confirmed by Andrade et al. (2022), who reported a statistically significant association between biofilm production and *agr* type, the accessory gene regulator (*agr*) quorum-sensing system, which plays a key role in *S. pseudintermedius* pathogenesis and resistance.

### Antibiotic resistance in MRSP

Currently, MRSP poses an animal health issue that is comparable to the public health concern due to methicillin-resistant *S. aureus* (MRSA) in human medicine. In this context, the emergence in dogs of multidrug-resistant MRSP has become a serious veterinary challenge worldwide, limiting the treatment options and representing a relevant threat to small animal therapy.

The rapid evolution of MRSP has changed its population structure, potentially influenced by the rising international movement of dogs for trade and recreational purposes, as well as by local antimicrobial usage practices, which may explain the geographical variations in MRSP lineages. To date, the noteworthy selective pressure exerted by the widespread usage of β-lactams in small animal veterinary medicine appears to be the main driver to MRSP selection and dissemination. Indeed, β-lactams account for approximately 70% of antimicrobial consumption in the veterinary sector according to various national surveys and clinical studies (Hur et al. 2020; Chirollo et al. 2021; Glavind et al. 2022).

A systematic review has previously shown that the resistance patterns of MRSP strains can differ significantly depending on their clonal lineage (Pires Dos Santos et al. 2016), highlighting the importance of understanding the genetic characteristics and antimicrobial susceptibility profiles of the isolates for effective treatment decisions. Notably, at the time this review was published, resistance to enrofloxacin, gentamicin, and chloramphenicol was relatively infrequent in ST258 in comparison to ST71 and ST45, which exhibited significantly higher rates of resistance to these antibiotics (Pires Dos Santos et al. 2016). These data suggest that the empirical choice of antimicrobial therapy for MRSP infections should take into account the regional prevalence of clonal type(s).

Oxacillin is used as a surrogate drug for the predicting of clinical efficacy of β-lactam antibiotics for treatment of *S. pseudintermedius* infections (Bemis et al. 2009; Skov et al. 2020). The current CLSI guidelines recommend that *S. pseudintermedius* isolates resistant to oxacillin should be reported as resistant to all β-lactams, without clinical findings (CLSI, 2020). Interestingly, Wegener et al. (2020) demonstrated in vitro susceptibility to amoxicillin/clavulanate and cephalothin in MRSP strains belonging to specific clonal complexes. On the contrary, high level of resistance to these β-lactams were observed among the ST71 carrying the SCC*mec*II-III (Wegener et al. 2020). The latter observation might be a possible explanation of the spread of this clone worldwide because of the high selective pressure exerted by the wide use of β-lactams in small animal veterinary practice.

The in vitro data published by Wegener et al. (2020) have potential clinical relevance, since amoxicillin-clavulanate and first generation cephalosporins are first-line antibiotics in the treatment of *S. pseudintermedius* pyoderma in dogs (Hillier et al. 2014). In this context, Pirolo et al. (2023) described the influence that penicillin-binding protein (PBP) mutations have on in vitro oxacillin MICs and consequently on susceptibility to β-lactam antibiotics in canine MRSP. In particular, the authors demonstrated that low-level MRSP (oxacillin MIC < 4 mg/L) cannot be classified as resistant to all β-lactams, since all tested MRSP resulted to be susceptible to cefalexin based on MIC and time kill testing, independently from the tested sequence type. In addition, to back up the obtained results, Pirolo et al. (2023) performed also a retrospective clinical study on dogs infected by low-level MRSP, highlighting, in these patients, a successful treatment with β-lactam antibiotics alone or in association with an aseptic topical therapy. Therefore, this data underlines the need of revising clinical β-lactams breakpoints, in order to reduce the prescription of critically important antimicrobials as fluoroquinolones as well as antimicrobials not approved for veterinary medicine, which should be reserved only for humans.

Multidrug-resistance in MRSP includes resistance to tetracyclines, macrolides, lincosamides, aminoglycosides, streptogramins, sulfamethoxazole-trimethoprim, and fluoroquinolones, as reported worldwide (Osland et al. 2012; Quitoco et al. 2013; Haenni et al. 2014; Grönthal et al. 2017; Worthing et al. 2018; Nocera et al.,2020b, c; Penna et al. 2022; Viegas et al. 2022; Morais et al. 2023; Sawhney et al. 2023). Infections caused by multidrug-resistant MRSP strains frequently lead to the use of alternative antibiotics in dogs, like mupirocin (Frank and Loeffler 2012; McCarthy et al. 2015). Even though mupirocin is not licensed for dogs in many European countries and its administration should be avoided according to AMEG categorization, given its use in humans, veterinarians may resort to its off-label use for topical treatments. Nowadays, resistance to mupirocin has been occasionally described in MRSP strains, also because its susceptibility is rarely evaluated for animal *Staphylococcus* spp isolates (Kizerwetter-Świda et al. 2019). Mupirocin resistance has been well characterized in *S. aureus*, and it is classified in low-level mupirocin resistance (MIC values range from ≥ 8 µg/ml to 256 µg/ml) caused by a point mutation in the chromosomal *ileS* gene, and high-level mupirocin resistance (MIC values ≥ 512 µg/ml) linked to the plasmid-encoded *ile*S2 gene (Godbeer et al. 2014). Many European and non-European studies described the occasional occurrence of MRSP and MSSP strains showing high-level mupirocin resistance (Godbeer et al. 2014; Bean et al. 2016; Park et al. 2018; Kizerwetter-Świda et al. 2019).

Consequently, when antibiotics are prescribed to small animals, it is important to remember that most of antibiotics are used also in human medicine, either as identical or related molecules, so they should be prescribed and administered wisely and whenever possible supported by antimicrobial susceptibility testing in accordance with national and international guidelines and regulations on use of antibiotics in animals. In any case, it is necessary to promote and to implement effective antimicrobial stewardship interventions in veterinary medicine. Overall, the monitoring of antibiotic resistance profiles results to be relevant to preserve the efficacy of available antibiotics for a long time.

### New antimicrobial treatment strategies and alternatives to antibiotics

The increasing spread of multidrug-resistant MRSP and MSSP strains and the lack of effective, new, conventional antibiotics highlight the limited therapeutic possibilities and the need of new strategies to control and treat infections.

A potential non-antibiotic, anti-infective, alternative therapeutic approach for treating canine multidrug-resistant infections is represented by DIBI, a novel purpose-designed water-soluble hydroxypyridinone-containing iron-chelating antimicrobial 9 kDa polymer, whose antimicrobial activity is explicated by depleting iron, which is required for bacterial growth. Precisely, DIBI is a novel highly selective iron chelator belonging to the synthetic hydroxypyridinone-class chelators. Indeed, DIBI antimicrobial activity is performed by intracellularly sequestration of iron to limit its availability to the bacteria, thus counteracting the bacterial siderophores, low-molecular-weight and high affinity iron chelators, in harvesting iron from the host and /or the environment (Ang et al. 2018). Nocera et al. (2022) showed low MIC values of DIBI (2 µg/mL) in four clinical multidrug-resistant MSSP strains along with broad antimicrobial and anti-infective activities, which were independent of the antimicrobial resistance profile of each tested strain (Nocera et al. 2022). Previous studies have shown that DIBI is also active against other Gram-positive as well as Gram-negative bacteria (*S. aureus* and *Acinetobacter baumannii*) (Ang et al. 2018; Parquet et al. 2018, 2019) and fungi (*Candida albicans*) (Ang et al. 2018; Savage et al. 2018), thereby proving to be a useful drug for management of mixed infections. DIBI has been purposely developed to bolster the existing innate iron withdrawal host defenses and has been shown to be orally and systemically non-toxic when administrated at repeated high dosages as well as to the ear canals of healthy dogs (Holbein et al. 2021).

Alternative treatments for MRSP infections include essential oils (EOs) and antimicrobial peptides (AMPs).

EOs are known to possess antiseptic, antibacterial, antiviral, antioxidant, anti-parasitic, antifungal, and insecticidal activities. Therefore, EOs can serve as a powerful tool to counteract the bacterial resistance (Chouhan et al. 2017). In this regard, Song et al. (2013) demonstrated that manuka oil (*Leptospermum scoparium*) had an excellent in vitro antimicrobial activity against MRSP and MSSP strains isolated from dogs suffering from superficial pyoderma and otitis. Besides *L. scoparium*, also for *Cinnamomum zeylanicum*, *Melissa officinalis*, *Satureja montana*, and *Cymbopogon citratus* EOs a promising in vitro antibacterial activity has been reported against both canine MRSP and MSSP strains (Nocera et al. 2020a). Moreover, it has been also demonstrated that the bioactive fungal compound harzianic acid, the main metabolite produced by strains ET45 and E45 of *Trichoderma harzianum*, is a potential antimicrobial and antibiofilm agent against strains of *S. pseudintermedius*-associated canine skin diseases (De Filippis et al. 2020). Brown et al. (2020) showed that manuka honey not only had a relevant antibacterial activity against multidrug-resistant *S. pseudintermedius* strains but also a synergic activity with four antibiotics (gentamicin, tetracycline, penicillin, chloramphenicol).

In addition to their antimicrobial properties, AMPs have immunomodulating effects that protect the host from pathogen infections (Liu et al. 2021). Nowadays, they are considered a valid alternative to conventional antimicrobials mainly for two reasons: *i*) the broad-spectrum of activity; *ii*) the low propensity to induce resistance development and selection of MRSP. Currently, AMPs are promisingly used for the topical treatment of canine infection as shampoos, foams, and ear gels, containing or plant extracts able to promote the production of endogenous AMPs by the canine skin (Santoro et al. 2017) or a cyclic β-sheet synthetic peptide (AMP2041), possessing a broad-spectrum antimicrobial activity (Cabassi et al. 2013, 2017).

In a Polish study (Jarosiewicz et al. 2020), seven AMPs (aurein 1.2, CAMEL, citropin 1.1, protegrin-1, pexiganan, temporin A, and uperin 3.6), with a well-known antibacterial activity against human pathogens, were tested against *S. pseudintermedius*-associated canine pyoderma. Tested AMPs were extensively effective against both canine multidrug-resistant MRSP and MSSP. The most active peptide resulted to be uperin 3.6 with MIC values of 2 µg/mL, demonstrating that uperin 3.6 could be considered as a potential antibacterial agent, especially adequate for the treatment of multidrug-resistant *S. pseudintermedius* infections (Jarosiewicz et al. 2020). In Italy, Bellavita et al. (2020) demonstrated that peptide 8 derived from Temporin L, an antimicrobial peptide isolated from the skin of *Rana temporaria*, was able not only to inhibit the growth of both canine MRSP and MSSP clinical strains with MIC values of 6.25 µM and 1.56 µM, respectively, but showed also a good antibiofilm activity. Intriguingly, peptide 8 enhanced oxacillin activity, inducing an increased susceptibility of MRSP to this antibiotic. In addition, being effective also against *Malassezia pachydermatis*, peptide 8 might represent a promising therapeutic alternative in the treatment of interkingdom polymicrobial infections (Bellavita et al. 2020).

In vitro antibacterial and antibiofilm activity was also described for two newly discovered AMPs allomyrinasin and andricin B, which possessed a strong bioactivity against *S. pseudintermedius* strains isolated from canine pyoderma samples, showing both MIC values of 8 µg/mL (Tang et al. 2021). The antimicrobial activity of these two cationic AMPs was explicated through the disruption of microbial cell membrane. Furthermore, these peptides displayed, when combined, a synergistic activity against *S. pseudintermedius* with a FIC index of 0.3125 and a good anti-inflammatory activity, above all allomyrinasin. Thus, allomyrinasin and andricin B might provide an ideal approach for treating canine skin infections, particularly for their high stability and their potent antibacterial ability in vitro (Tang et al. 2021). Despite the clear and good potential AMPs have for topical therapy, some doubts remain about their efficacy when used for systemic treatment.

The recent evolution of nanotechnology has accelerated the development of metal nanoparticles, and among them, the most successful antimicrobial agents are represented by silver nanoparticles (AgNPs) also for their low toxicity to mammalian cells (Franci et al. 2015). Moreover, AgNPs are further divided into two groups depending on their shape: Ag nanospheres (AgNSs) and anisotropic Ag nanoparticles (without a spherical shape). Currently, AgNSs are commonly used as antimicrobial agents, but for the anisotropic AgNPs, stronger antibacterial and antibiofilm abilities have been reported against canine *S. pseudintermedius* clinical strains, due to their particle size and shape (Meroni et al. 2020; Thammawithan et al. 2021a). Interestingly, Thammawithan et al. (2021a) found that the antimicrobial activity of AgNPs against MRSP was slightly lower than that against MSSP. Regarding this finding, authors hypothesized that it might be linked to some cell components, or a resistance mechanism not found in MSSP (Thammawithan et al. 2021a). Furthermore, in another study antimicrobial and wound healing activities of anisotropic AgNPs gels were assessed in vivo by Thammawithan et al. (2021b), demonstrating that anisotropic AgNPs gel helps wound repair accompanied by a reduction of scar formation.

Another potential therapeutic alternative against MRSP and MSSP is represented by bacteriophages that specifically infect bacterial hosts, even though it is important to consider that the major concern on phage therapy is the transmission possibility of the antimicrobial-resistant- or virulence-related genes (Penadés et al. 2015). However, there are also other concerns associated with the delivery of phages to the site of infection, such as the rapid emergence of bacterial resistance to them, and the consequent need to prepare and update phages libraries to counteract the emergence of resistance. A Danish study provided insights into the genetic diversity and biology of *S. pseudintermedius* temperate phages, which could be further developed for topical therapy of MRSP skin and wound infections (Moodley et al. 2019). In that report the ability of the tested phages to lyse especially MRSP with respect to MSSP strains was described (Moodley et al. 2019). The study also highlighted the difficulty in isolating lytic phages, which would be more suitable than temperate phages for therapeutic applications. Subsequently, two novel bacteriophages as well as their cocktail have been studied to evaluate their capability to prevent biofilm formation at low doses and to degrade biofilm at high doses (Kim et al. 2021). Few years ago, the first lytic *S. pseudintermedius* phages were discovered by Hernandez Santos et al. (2022). Further research is warranted to evaluate the efficacy of these phages using murine models of canine pyoderma, such as the murine cutaneous-infection model described firstly by Richards et al. (2018) to investigate the role of SpsD and SpsL, two *S. pseudintermedius* cell wall-associated proteins, in skin disease pathogenesis. In addition, another study (Bünsow et al. 2021) used a panel of genetically engineered MRSP variants and a mouse abscess model to identify *S. pseudintermedius* major secreted nuclease (NucB) and adenosine synthase A (AdsA) and to provide new insights on *S. pseudintermedius* virulence and immune evasive abilities. This latter study suggested the knowledge of the involved pathway during an invasive disease useful to better define the development of new therapeutic strategies. Only an in-depth knowledge of MRSP pathogenesis may help in the development of new therapeutic strategies as valid weapons to combat and control MRSP infections. However, the thorough investigation of new therapeutic alternative in relation to known and commonly circulating clones remains a highly intriguing area for future studies.

## Conclusions

This narrative review highlights a global evolution of the MRSP population, with the emergence of new antimicrobial-resistant clones both in European and non-European countries, which have been replacing the major clonal lineages, such as ST71. The increasing spread of multidrug-resistant MRSP strains represents not only a worrying threat to animal health for their propensity to dog-to-dog transmission, but also a concern for human health due to their potential transmission to people, particularly veterinarians and pet owners.

Furthermore, the limited therapeutic options and the critical need to treat infections caused by bacterial clones with multidrug resistance profiles have addressed many scientists to develop new therapeutic strategies effective against difficult-to-treat multidrug-resistant pathogens, as *S. pseudintermedius*. However, believing that the antimicrobial resistance issue can be solely solved by the development of new therapeutic strategies is illusory.

For all the above reasons, this review displays the need for periodic studies on monitoring and epidemiological surveillance, to better understand how such an important pathogen as MRSP is capable of rapidly evolving, as well as the importance of preventing the spread of MRSP in canine population and veterinary settings, including stewardship programs for rational use of available antimicrobials and the implementation of adequate preventive measures. Finally, it is of critical importance to acquire a more comprehensive understanding of the molecular, evolutionary, and ecological mechanisms governing the spread of difficult-to-treat multidrug-resistant pathogens.

## Data Availability

No datasets were generated or analysed during the current study.
